# Plexin-Semaphorin Signaling Modifies Neuromuscular Defects in a *Drosophila* Model of Peripheral Neuropathy

**DOI:** 10.3389/fnmol.2018.00055

**Published:** 2018-02-22

**Authors:** Stuart J. Grice, James N. Sleigh, M. Zameel Cader

**Affiliations:** ^1^MRC Functional Genomics Unit, Department of Physiology, Anatomy and Genetics, University of Oxford, Oxford, United Kingdom; ^2^Sobell Department of Motor Neuroscience and Movement Disorders, Institute of Neurology, University College London, London, United Kingdom; ^3^MRC Weatherall Institute of Molecular Medicine, John Radcliffe Hospital, University of Oxford, Oxford, United Kingdom

**Keywords:** axonal guidance, aminoacyl-tRNA synthetase (ARS), Charcot-Marie-Tooth disease type 2D (CMT2D), distal spinal muscular atrophy type V (dSMA-V), *GARS*, glycyl-tRNA synthetase, neuromuscular disease, neurodevelopment

## Abstract

Dominant mutations in *GARS*, encoding the ubiquitous enzyme glycyl-tRNA synthetase (GlyRS), cause peripheral nerve degeneration and Charcot-Marie-Tooth disease type 2D (CMT2D). This genetic disorder exemplifies a recurring paradigm in neurodegeneration, in which mutations in essential genes cause selective degeneration of the nervous system. Recent evidence suggests that the mechanism underlying CMT2D involves extracellular neomorphic binding of mutant GlyRS to neuronally-expressed proteins. Consistent with this, our previous studies indicate a non-cell autonomous mechanism, whereby mutant GlyRS is secreted and interacts with the neuromuscular junction (NMJ). In this *Drosophila* model for CMT2D, we have previously shown that mutant *gars* expression decreases viability and larval motor function, and causes a concurrent build-up of mutant GlyRS at the larval neuromuscular presynapse. Here, we report additional phenotypes that closely mimic the axonal branching defects of *Drosophila* plexin transmembrane receptor mutants, implying interference of plexin signaling in *gars* mutants. Individual dosage reduction of two *Drosophila* Plexins, *plexin A (plexA)* and *B (plexB)* enhances and represses the viability and larval motor defects caused by mutant GlyRS, respectively. However, we find plexB levels, but not plexA levels, modify mutant GlyRS association with the presynaptic membrane. Furthermore, increasing availability of the plexB ligand, Semaphorin-2a (Sema2a), alleviates the pathology and the build-up of mutant GlyRS, suggesting competition for plexB binding may be occurring between these two ligands. This toxic gain-of-function and subversion of neurodevelopmental processes indicate that signaling pathways governing axonal guidance could be integral to neuropathology and may underlie the non-cell autonomous CMT2D mechanism.

## Introduction

Dominant mutations in the glycyl-tRNA synthetase (GlyRS) gene, *GARS*, cause Charcot-Marie-Tooth disease type 2D (CMT2D; Antonellis et al., 2003). CMT is a broad group of genetically heterogeneous peripheral neuropathies that present with progressive motor and sensory degeneration. Type 1/Demyelinating CMT is typified by demyelination leading to reduced nerve conduction velocities (NCVs), Type 2/Axonal CMTs display axonal loss with relatively normal NCVs, while intermediate forms share clinical features of the two. *GARS* is one of several aminoacyl-tRNA synthetase (ARS) genes linked to CMT (Jordanova et al., 2006; Latour et al., 2010; McLaughlin et al., 2010; Gonzalez et al., 2013; Vester et al., 2013). In total, there are 37 human ARS proteins, which catalyze the covalent linkage of specific amino acids to their partner transfer RNAs (tRNAs; Pang et al., 2014). As GlyRS, and ultimately glycine aminoacylation, is essential in almost all cell types (Antonellis et al., 2003; Alexandrova et al., 2015), the neuronal specificity of the disease is puzzling. A number of potential mechanisms have been hypothesized (Motley et al., 2010; Niehues et al., 2015; Storkebaum, 2016), although the exact pathological processes remain unknown. Notwithstanding, cellular and mouse studies suggest that CMT2D is caused by a gain-of-function in mutant GlyRS rather than *GARS* haploinsufficiency, or loss of canonical glycine aminoacylation or a secondary non-canonical function (Seburn et al., 2006; Nangle et al., 2007; Xie et al., 2007; Achilli et al., 2009; Motley et al., 2011).

Numerous mutations in *GARS* have been shown to cause a similar conformational change to GlyRS, leading to a structure that can bind to erroneous targets (He et al., 2011). Given that GlyRS is secreted (Park et al., 2012; Grice et al., 2015b; He et al., 2015), these mis-interactions are not necessarily restricted to within cells. Indeed, recent studies have shown that neomorphic regions in mutant GlyRS facilitate aberrant, extracellular interactions with Neuropilin 1 (Nrp1; He et al., 2015) and the tropomyosin receptor kinase (Trk) receptors (Sleigh et al., 2017a). In conjunction with plexins and other co-receptors, Nrp1 binds to extracellular signaling proteins such as the semaphorins and vascular endothelial growth factor (VEGF), and is part of cell-cell communication that governs the processes guiding the development of both the nervous and vascular systems (Neufeld et al., 2002; Supplementary Figure S1). Similarly, the Trk receptors bind to target-secreted, extracellular growth factors, and play a key role in the development, differentiation and survival of sensory nerves (Lindsay, 1996). The interaction of mutant GlyRS with Nrp1 selectively antagonizes VEGF signaling (He et al., 2015), preferentially impacting the nervous, but not the vasculature, system (Sleigh et al., 2017b). Neomorphic interactions between mutant GlyRS and anomalous targets are thus likely to play an important role in the manifestation of CMT2D.

CMT2D patients generally present during adolescence with progressive muscle weakness in the distal limbs due to axon loss, while NCVs and myelination remain unperturbed (Antonellis et al., 2003; Sivakumar et al., 2005). In addition to axonal degeneration (Seburn et al., 2006; Achilli et al., 2009), CMT2D mice display denervation (Seburn et al., 2006; Achilli et al., 2009; Motley et al., 2011; Sleigh et al., 2014b) and impaired neurotransmission (Spaulding et al., 2016) at the neuromuscular junction (NMJ), which is preceded by defective synaptic development (Sleigh et al., 2014b).

In *Drosophila*, we have shown that mutant GlyRS is secreted by muscle and interacts with the NMJ (Grice et al., 2015b). This gain-of-function, non-cell autonomous mechanism causes both degenerative and developmental abnormalities in the *Drosophila* nervous system. The mechanisms underlying these defects remain unknown, but interactions with neuropilin-plexin-semaphorin signaling is an attractive possibility for a number of reasons. First, in vertebrates, mutant GlyRS binds to and interferes with the canonical function of the plexin-interacting protein Nrp1. Second, plexin-semaphorin signaling controls processes perturbed in a number of CMT2D animal models (outlined above) including neurotransmission (Carrillo et al., 2010; Orr et al., 2017), synaptic establishment and maturation (Hu et al., 2001; Ayoob et al., 2006; Berke and Keshishian, 2011), and active zone protein dynamics (Orr et al., 2017). Finally, in this report, characterization of flies expressing mutant *gars* identified phenotypes that mimic those associated with perturbed plexin-semaphorin signaling. Though no direct *Drosophila* ortholog of Nrp1 has been identified, the fly has two plexin genes, *plexin A* (*plexA*; Winberg et al., 1998) and *plexin B* (*plexB*; Hu et al., 2001), which independently bind to different semaphorin classes and are critical to the process of axon guidance (Schuster et al., 1996; Hu et al., 2001), retrograde signaling at the synapse, and synaptic plasticity (Orr et al., 2017).

As mentioned above, we now report that mutant GlyRS expression mimics the axonal branching defects generated by loss-of-function mutations in the two fly plexins, *plexA* and *plexB*. Moreover, we show that dosage reduction of *plexA* and *plexB* enhances and represses the viability and motor function defects caused by mutant *gars* expression, respectively. *plexA* and *plexB* dosage changes also modify adherent axonal protein localization, whilst reduction of *plexB* ameliorates the toxic build-up of GlyRS at the synapse. The centrality of PlexB signaling to mutant GlyRS toxicity is further highlighted by the observation that overexpression of the PlexB ligand, Semaphorin-2a (Sema2a), suppresses mutant GlyRS synaptic binding and associated neuropathology. This reveals an important role for plexins in mutant GlyRS pathology and suggests a possible mechanism by which mutant GlyRS can exert non-cell autonomous effects.

## Materials and Methods

### General Methods

Reagents were obtained from Sigma-Aldrich unless otherwise stated. All *Drosophila* stocks were cultured on standard molasses/maize meal and agar medium in plastic vials or bottles at 20 or 25°C. The *gars*^P234KY^ and *gars*^wt^ stocks have been previously described (Grice et al., 2015b). *1032-GAL4*, *elav-GAL4*, *MHC-GAL4*, the plexA loss-of-function mutant *plexA*^MB09499^, *UAS-plexA* and the plexB loss-of-function mutant *plexB*^KG00878^, were obtained from the Bloomington *Drosophila* Stock Center at Indiana University, and Alex Kolodkin generously shared the *UAS-Sema-1a, UAS-Sema-2a-TM-GFP*, and *UAS-plexB-myc* lines.

### *Drosophila* Viability and Behavioral Assays

Adult viability assays were conducted by crossing *GAL4* driver stocks to lines harboring *gars* overexpression or to *w*^1118^ for controls. Embryos were then counted, lined on apple juice plates with yeast, and larval development noted. Fresh yeast was added daily. Two days post-eclosion the number of surviving adult flies was counted for each genotype. Hundred flies per genotype were scored over three independent experiments. Measurement of motor function involved placing individual age-matched third instar larvae at the center of a 0.7% (w/v) agar plate and counting the forward body wall contractions exhibited in 2 min. Twenty flies per genotype were scored.

### *Drosophila* Larval NMJ Analysis

Larvae were reared, dissected and processed as previously described with analysis of hemisegment A2 (Schuster et al., 1996; Grice et al., 2015a,b), and at least 23 NMJs per genotype per stage were scored. Ectopic branch contact analysis was performed using L3 larval hemisegments A1 to A3, and 14 flies per genotype were scored. For axonal bruchpilot (Brp) localization studies, the transverse nerve (TN) section bypassing muscle 6 and 7 in hemisegments A2 and A3 was used. For immunohistochemistry, anti-discs large (DLG, DSHB), anti-HRP (Jackson), anti-Bruchpilot (DSHB) and anti-HA (Santa Cruz) were all used at 1/100 in combination with secondary antibodies, AlexaFluor 488 goat anti-rabbit and AlexaFluor 633 goat anti-mouse (Invitrogen) at 1/1000. Z-stacks were taken using a Leica SP5 laser confocal microscope and analysis performed using ImageJ and Photoshop (Adobe).

### Quantitative RT-PCR (qPCR)

RNA was extracted from dissected L3 larval ventral nerve cords using an RNeasy Mini Kit (QIAGEN), as per the manufacturer’s instructions, and reverse transcribed as published previously (Sleigh et al., 2014a). cDNA was diluted in water (1/10) before mRNA levels were assessed by qPCR in 20 μl reactions using SYBR Green (Applied Biosystems) and a StepOnePlus real-time PCR machine (Applied Biosystems). Relative gene expression was assessed as previously described (Lee et al., 2008; Sleigh et al., 2014a). Primer sequences were as follows: *plexA* forward 5′-GCT CGT CGT TGA CAA AAT TCG-3′ and reverse 5′-TAA ATG CGA CCC AGA TTG GTG C-3; *plexB* forward 5′-GCG TTT TCA TCG TTA GCA GC-3′and reverse 5′-CAT TCG ACA AGG AGC CTG C-3′. Three technical replicates per reaction were performed, with each of three biological replicates, and expression was normalized to *rp42*.

### Statistical Analysis

When normally distributed, datasets were statistically analyzed using either an unpaired *t*-test with Welch’s correction or a one-way analysis of variance with Bonferroni’s multiple comparison test. If the data did not pass normality testing, the non-parametric Mann–Whitney *U* test or Kruskal–Wallis test with Dunn’s multiple comparison test was used. Linear regression analyses were performed to determine the correlation between synaptic accumulation of mutant GlyRS and neuropathological features of the CMT2D model. GraphPad Prism 5 software was used for all statistical analyses.

## Results

### *gars*^P234KY^ Expression Alters Neurodevelopmental Wiring

We use a *Drosophila* larval model for CMT2D (Grice et al., 2015b), and concentrate on neuronal phenotypes in the neuromuscular system caused by the mutant *gars* expression. Both developmental and degenerative defects have been observed in *Drosophila* CMT2D models (Chihara et al., 2007; Ermanoska et al., 2014; Grice et al., 2015b; Niehues et al., 2015); however, the causative mechanisms for many of these phenotypes have not been identified. We have previously shown that expression of mutant GlyRS in flies leads to motor deficits and progressive NMJ denervation, mirroring CMT2D mice (Sleigh et al., 2014b, 2017a; Spaulding et al., 2016), as well as presynaptic accumulation of mutant GlyRS (Grice et al., 2015b). In addition, we find that the neuronal toxicity is, at least in part, non-cell autonomous (Grice et al., 2015b). Furthermore, defects in synaptic establishment, maturation, function and maintenance have been observed in invertebrate (Grice et al., 2015b) and vertebrate *Gars* neuropathy models (Sleigh et al., 2014b; Spaulding et al., 2016).

**Figure 1:**
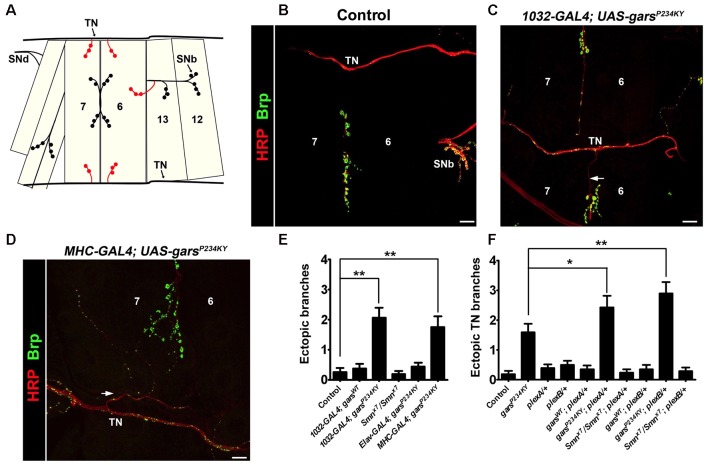
*gars*^P234KY^ expression phenocopies plexin mutants and subverts plexin-mediated axonal branching. **(A)** Schematic of the larval ventral body wall muscles in one hemisegment, showing the transverse nerve (TN), and branches of segmental nerves b (SNb) and d (SNd). The ectopic synaptic contacts often observed on muscles 6 and 7 in *gars*^P234KY^ flies are shown in red. **(B)** In wild-type flies, SNb innervates muscle 12, while the TN entirely bypasses muscles 6, 7, 12 and 13. **(C,D)** Ectopic axons (arrows) and synaptic contacts can be observed from the SNb and the TN when *gars*^P234KY^ is expressed ubiquitously **(C)** or in muscle **(D)**, phenocopying loss-of-function *plexB* homozygotes (Carrillo et al., 2010). **(E)** Ubiquitous (*1032-GAL4*) and muscle (*MHC-GAL4*) *gars*^P234KY^ expression, but not mutant neuronal (*elav-GAL4*) or ubiquitous wild-type *gars* expression, lead to an increased number of ectopic contacts in L3 larvae. This is not seen in a model of spinal muscular atrophy (*smn*^x7^/*smn*^x7^), a second, unrelated neuromuscular condition. Ectopic branches are scored from both the TN and the SNb nerve. **(F)** Expressing *gars*^P234KY^ with a ubiquitous driver in either a *plexA* or *plexB* heterozygous knockout background significantly enhances the branching defects from the TN nerve. *P < 0.05, **P < 0.01, Dunn’s multiple comparison test. N.b., *gars*^P234KY^ is not expressed in the following flies: control, *Smn*^x7^/*Smn*^x7^, *plexA/+*, *plexB/+*, *Smn*^x7^/Smn^x7^; *plexA/+* and *Smn*^x7^/Smn^x7^; *plexB/+* **(E,F)**. Scale bars = 10 μm. For all experiments, *n* > 16 larvae per genotype. Error bars represent ± standard error of the mean (SEM). See also Supplementary Figure S2.

We now report a novel CMT2D phenotype of defective synaptic wiring in the *Drosophila* neuromuscular system. The *Drosophila* larval abdominal ventrolateral muscles 6 and 7 are innervated in a stereotyped manner by defasciculated segmental nerve d (SNd; Figures 1A,B). Other proximal nerves, such as the TN and SNb, normally bypass these muscles. In wild-type flies, SNb innervates the adjacent muscle 12, while the TN entirely circumvents muscles 6, 7, 12 and 13. When a mutant form of GlyRS is ubiquitously expressed using the GAL4-UAS system (*1032-GAL4* driver), ectopic synaptic contacts from both the TN (Figures 1B,C; Supplementary Figures S2C,D) and SNb (Supplementary Figure S2B) are often observed on muscles 6 and 7 in *gars*^P234KY^ flies. Consistent with the non-cell autonomous disease mechanism we have previously identified (Grice et al., 2015b), this occurs when *gars*^P234KY^ is expressed from muscle (*MHC-GAL4*; Figure 1D), but not neurons (*elav-GAL4*), nor when wild-type *gars* is ubiquitously expressed using the same driver (Figure 1E). This indicates that mutant GlyRS disrupts normal axonal guidance, and that this possibly occurs through cell-to-cell signaling rather than disturbance of inherent neuronal pathways. We did not see comparable defects in a model of spinal muscular atrophy (SMA; *Smn*^x7^/*Smn*^x7^), suggesting that these defects are not general to all *Drosophila* models of motor neuron degeneration.

### Heterozygous *plexA* and *plexB* Mutations Enhance *gars*^*P234KY*^ Axonal Branching Defects

A recent study has shown that mutated GlyRS binds to the membrane-bound protein Nrp1 (He et al., 2015), which acts as a co-receptor with mammalian Plexin A for semaphorin signaling (Tran et al., 2007). Whilst there is no direct Nrp1 ortholog, the fly has two plexin orthologs, *plexA* (Winberg et al., 1998) and *plexB* (Hu et al., 2001), which independently bind to different semaphorin classes. Due to their role in axonal attraction and repulsion, knock-down of the *Drosophila* plexins drives diverse axon guidance defects in the embryo, while total loss of the plexin proteins leads to fly lethality (Winberg et al., 1998; Hu et al., 2001; Ayoob et al., 2006). Intriguingly, the CMT2D model presented here (i.e., mutant *gars*-expressing flies), phenocopies the wiring defects of both *plexA* and *plexB* loss-of-function mutants (Figures 1A–E). We therefore looked to see if alterations in plexin levels modify the axonal guidance defects observed when *gars*^P234KY^ is expressed. To reduce plexin levels in mutant *gars* flies, we crossed heterozygous loss-of-function mutants of both *plexA* (*plexA*^MB09499^) and *plexB* (*plexB*^KG00878^) to flies ubiquitously expressing *gars*^P234KY^, and re-scored the number of ectopic contacts occurring between the TN and muscles 6 and 7. We found that heterozygous reduction of both *plexA* and *plexB* enhanced the ectopic branching defects observed in mutant GlyRS flies (Figure 1F), suggesting that *gars*^P234KY^ expression interferes with plexin-mediated axonal guidance. Again, we did not see any modifications in the SMA model (*Smn*^x7^/*Smn*^x7^), suggesting that the enhancements are specific to the CMT2D model.

### Differential Effects of PlexA and B on *gars*^*P234KY*^ Lethality and Motor Dysfunction

Mutant GlyRS causes both larval and adult phenotypes in the fly (Ermanoska et al., 2014; Grice et al., 2015b; Niehues et al., 2015); however, the axonal branching defects in both plexin and *gars*^P234KY^ mutants are likely to originate during embryonic development and larval growth. We therefore assessed *plexA* and *plexB* expression in larval and adult nervous systems using quantitative RT-PCR (qPCR). The RNA of both genes was detected in the larval central nervous system, and the adult brain and ventral nerve cord (Supplementary Figure S3), suggesting that both plexins function throughout life.

**Figure 2:**
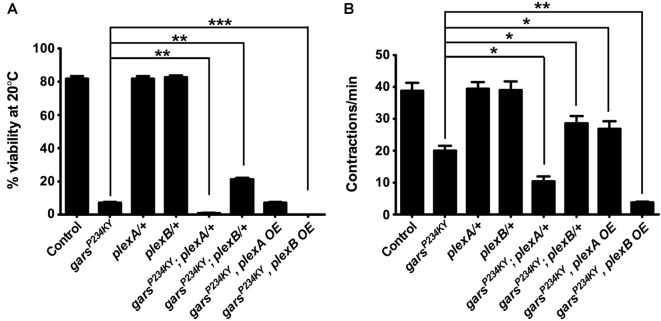
Plexin mutants modify glycyl-tRNA synthetase (GlyRS)^P234KY^-associated viability and motor defects. **(A)** Expressing *gars*^P234KY^ with a ubiquitous driver (*1032-GAL4*) in a *plexB* heterozygous loss-of-function background significantly alleviates adult viability defects, while *plexB* overexpression reciprocally causes full lethality. Contrastingly, *gars*^P234KY^ expression in a *plexA* loss-of-function background leads to a decrease in viability, while its overexpression has no effect.** (B)** *gars*^P234KY^ expression in *plexB* heterozygous larvae partially rescues muscle contractions, whilst *gars*^P234KY^ expression in a *plexA* background enhanced the motor defect. *plexA* overexpression partially restores movement, while *plexB* overexpression enhances the motor defect. The larval motor defects are thus analogous to the adult viability defects in **(A)**. *P < 0.05, **P < 0.01, ***P < 0.001 Dunn’s multiple comparison test. N.b., *gars*^P234KY^ is not expressed in the *plexA/+* and *plexB/+*, control flies (third and fourth bars from the *y*-axes, respectively). For viability assays, *n* > 100 flies per genotype; for contraction assays, *n* > 20 larvae per genotype. Error bars represent ± SEM.

We next looked to see how modifying plexin levels impacts the adult lethality and larval motor defects when *gars*^P234KY^ is expressed ubiquitously using *1032-GAL4* (going forward referred to as *gars*^P234KY^ flies). Ubiquitous expression of *gars*^P234KY^ significantly reduced adult viability, with only 7% surviving to the adult stage. We found *plexA* and *plexB* reduction had opposing effects on the adult viability defect of *gars*^P234KY^ flies: reduction of *plexA* compounded the reduced viability (1% survival), whilst diminishing *plexB* levels significantly improved survival (21% survival). Alternatively, increasing *plexA* had no significant effect, while *plexB* overexpression reciprocally exacerbated the *gars*^P234KY^ viability phenotype, with no flies (0%) making it to the late pupal stage (Figure 2A). These results were corroborated by larval motor function testing (Figure 2B). When mutant *gars* is expressed, larval muscular contractions (a surrogate for larval motor function) decrease in number (~20 contractions/min are observed in the mutant compared to ~39 mean in controls). The defects in muscle function of mutant *gars*-expressing flies were worsened in *plexA* heterozygotes (~10 contractions/min) and marginally improved in plexA overexpressors (~27 contractions/min). *PlexB* reduction improved contraction frequency (~28 contractions/min) and *plexB* overexpression exacerbated it (~4 contractions/min). It should be noted that reduced PlexB levels enhanced the wiring defect (Figure 1F) but ameliorated the viability and motor phenotypes (Figure 2), suggesting that the neurodevelopmental branching defects do not contribute to the reduced viability of *gars*^P234KY^ flies and that mutant pathology is not due to loss of PlexB function. Due to the severe nature of the adult phenotypes, and given that the *gars*^P234KY^ flies surviving to adulthood die soon after eclosing, we did not perform movement assays in adults.

### Plexin Levels Differentially Modify Axonal Accumulation of the Active Zone Protein Bruchpilot in Mutant GlyRS Flies

**Figure 3:**
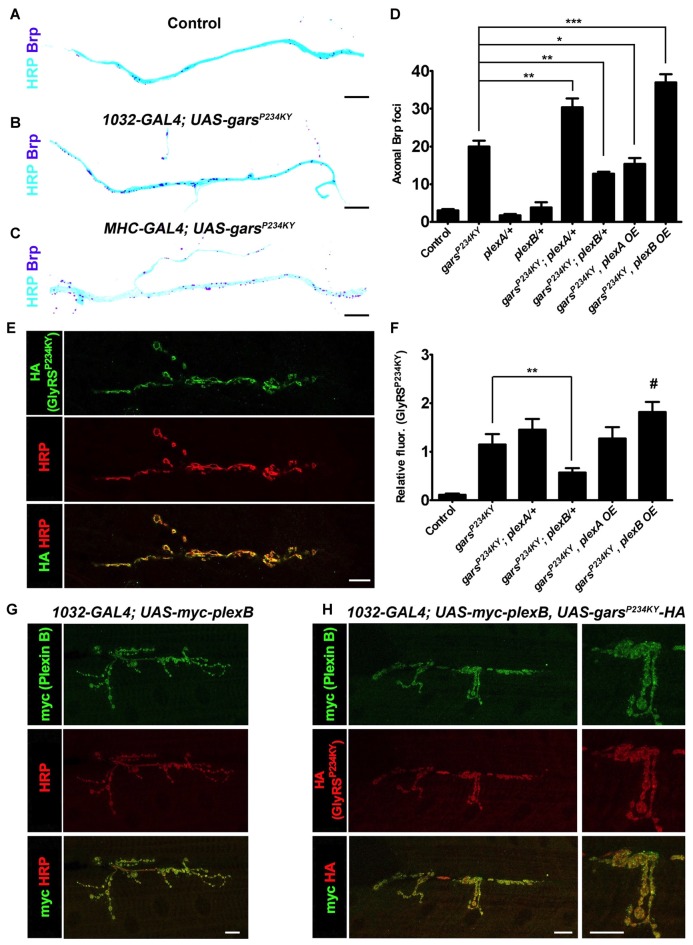
*plexA* and *plexB* dosage changes modify GlyRS^P234KY^-associated axonal defects and mutant GlyRS accumulation at the neuromuscular junction (NMJ). **(A–C)** Digitally inverted images of the TN showing increased axonal bruchpilot (Brp) compared to control **(A)** upon ubiquitous **(B)** and muscle-specific **(C)** *gars*^P234KY^ expression. **(D)** The build-up of Brp in axons of the TN caused by ubiquitous GlyRS^P234KY^ expression is modulated by alterations in PlexA or PlexB levels. Greater Brp accumulation is observed upon PlexA reduction and PlexB overexpression, whereas an increase in PlexA and a decrease in PlexB ameliorate Brp build-up. N.b., *gars*^P234KY^ is not expressed in the *plexA/+* and *plexB/+* control flies (third and fourth bars from the *y*-axis, respectively).** (E)** An example of GlyRS^P234KY^ (HA staining) accumulation at the presynapse when ubiquitously expressed. **(F)** GlyRS^P234KY^ accumulates at the neuronal membrane, and correlates with PlexB, but not PlexA, levels, i.e., increased and decreased abundance of PlexB causes increased and decreased mutant GlyRS build-up, respectively.** (G)** When expressed with a ubiquitous driver, PlexB localizes at the NMJ with the neuronal marker HRP.** (H)** PlexB and GlyRS^P234KY^ localize to the NMJ when co-expressed using a ubiquitous driver. *P < 0.05, **P < 0.01, ***P < 0.001 Dunn’s/Bonferroni’s multiple comparison test. *#*: displayed a significant increase when compared to the mutant GlyRS background alone (Mann-Whitney *U* test, *P* = 0.03). All experiments are performed expressing *gars*^P234KY^ with a ubiquitous driver (*1032-GAL4*). Scale bar = 10 μm. For all experiments, *n* > 20 larvae per genotype. Error bars represent ± SEM. See also Supplementary Figure S4.

We next determined how altered plexin levels modify additional key phenotypes of the *Drosophila* CMT2D model. First, we looked at the previously described axonal accumulation of the active zone-associated protein Brp (Figures 3A–D, Supplementary Figure S4; Grice et al., 2015b), which is a potential early indicator of neuropathology (Johnson et al., 2009) that may be caused by axonal transport defects (Goldstein et al., 2008). Both ubiquitous and muscle expression of mutant GlyRS lead to Brp protein accumulation in the axons of the peripheral nervous system. This appears to be driven via a non-cell autonomous mechanism as Brp build-up is not seen when mutant *gars* is expressed in neurons (Grice et al., 2015b). Quantification of the number of Brp-positive foci in the region of the TN adjacent to muscle 6 and 7 was performed in GlyRS mutant flies crossed with either the plexin heterozygous loss-of-function mutants, or the plexin overexpressors. In wild-type and control flies, Brp foci are limited, but can be observed in some axons (Figure 3A; ~3 axonal foci per section in controls). This number dramatically increases when mutant *gars* is expressed (~20 axonal foci per section). Concordant with the differential effects on viability (Figure 2), a reduction of *plexB* levels reduced Brp accumulation (~12 axonal foci per section), while *plexB* overexpression enhanced Brp accumulation (~36 axonal foci per section; Figure 3D). Conversely, Plexin A overexpression reduced the accumulation of axonal Brp in the GlyRS mutant flies, while *plexA* reduction enhanced the build-up.

### Plexin B Levels but Not Plexin A Modify Mutant GlyRS Accumulation at Neuromuscular Synapses

The neomorphic binding of mutant GlyRS to neuronal membrane proteins has been shown to be a potential mechanism for the toxic gain-of-function associated with CMT2D (He et al., 2015; Sleigh et al., 2017a). We therefore wanted to understand if alterations in plexin levels affect the accumulation of mutant GlyRS at the *Drosophila* neuromuscular synapse. We have previously reported that mutant but not wild-type GlyRS accumulates at the presynaptic NMJ in third instar larvae (Grice et al., 2015b; Figure 3E). This build-up coincided with NMJ defects described in the same report. We found that reducing *plexB* decreased the intensity of mutant GlyRS staining at the presynapse (51% decrease, Figure 3F). There was a trend for *plexB* overexpression to increase the build-up of mutant GlyRS at the presynapse, which was significantly greater when compared is isolation to the mutant GlyRS flies (35% increase Mann-Whitney *U* test, *P* = 0.03), suggestive of a reciprocal pattern associated with down- and up-regulation of Plexin B. No significant alterations were observed with *plexA* reduction or overexpression, suggesting that the synaptic accumulation of mutant GlyRS is observably modified by Plexin B alone. The expression patterns of plexins, are not well understood, due in part to a lack of reliable antibodies. We therefore overexpressed myc-tagged Plexin B and found that it localized to the presynaptic neuron (Figure 3G), where it co-stained with the mutant GlyRS protein (Figure 3H). The dependence of presynaptic GlyRS accumulation on Plexin B parallels the mammalian association of mutated GlyRS with Nrp1 (He et al., 2015).

### Overexpression of Sema2a, the PlexB Ligand, Suppresses Mutant GlyRS-linked Neuropathology

**Figure 4:**
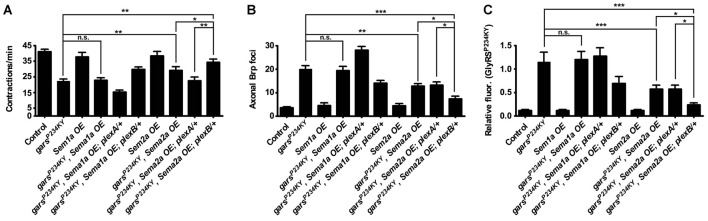
Overexpression of the Plexin B ligand Semaphorin-2a (Sema2a) suppresses mutant GlyRS-mediated neuropathology. **(A)** Expressing *Sema2a*, but not *Sema1a*, in *gars*^P234KY^ flies (using the muscle driver *MHC-GAL4*) significantly alleviates mutant motor defects (compare *gars*^P234KY^ with *gars*^P234KY^, *Sema2a OE*). In the *plexB* heterozygous loss-of-function background, this amelioration is further improved (compare *gars*^P234KY^, *Sema2a OE* with *gars*^P234KY^, *Sema2a OE; plexB/+*). In contrast, reduction of *plexA* levels abrogates the amelioration caused by *Sema2a* overexpression in *gars*^P234KY^ flies (compare *gars*^P234KY^, *Sema2a OE; plexA/+* with *gars*^P234KY^ and *gars*^P234KY^, *Sema2a OE*). **(B,C)** Consistent phenotype correlations were seen for both axonal Brp build-up **(B)** and synaptic accumulation of GlyRS^P234KY^ **(C)**. *P < 0.05, **P < 0.01, ***P < 0.001 Dunn’s/Bonferroni’s multiple comparison test. N.b., *gars*^P234KY^ is not expressed in the *Sema1a OE* and *Sema2a OE* control flies (third and seventh bars from the *y*-axes, respectively). All experiments are performed expressing *gars*^P234KY^ with a ubiquitous driver (*1032-GAL4*). For all experiments, *n* > 20 larvae per genotype. Error bars represent ± SEM. See also Supplementary Figure S3.

If presynaptic accumulation of mutant GlyRS and the associated neuromuscular defects are dependent on the aberrant binding of mutant GlyRS to plexins, we might expect that overexpression of the natural plexin ligands could supress the disease phenotypes through competitive binding. PlexA is the receptor for transmembrane semaphorin-1a (Sema1a), while PlexB is the main receptor for the secreted semaphorin Sema2a (Winberg et al., 1998; Ayoob et al., 2006). To mimic the non-cell autonomous effect, we expressed both Sema1a and Sema2a using a muscle-specific driver (Figure 4). Sema1a overexpression conferred no alteration in GlyRS^P234KY^ fly motor function (Figure 3A), axonal Brp accumulation (Figure 4B), or synaptic GlyRS build-up (Figure 4C). In addition, no further modifications were observed when Sema1a was overexpressed in the mutant *gars*^P234KY^; *plexA/+* and mutant *gars*^P234KY^; *plexB/+* backgrounds (Figure 4). This suggests that the mutant GlyRS pathology is not dependent on Sema1a-PlexA.

In stark contrast, Sema2a overexpression ameliorated the GlyRS^P234KY^ muscle contraction defect (Figure 4A, Supplementary Figure S4), as well as decreasing axonal Brp foci (Figure 4B) and aberrant synaptic GlyRS build-up (Figure 4C). Moreover, additive improvements in the three phenotypes occurred when mutant GlyRS and Sema2a were expressed in the plexB heterozygous mutant background (Figures 4A–C). Surprisingly, Sema2A overexpression eliminated the phenotypic enhancement in severity conferred by plexA heterozygosity. However, as a possible compensatory mechanism, *plexB* expression is modestly increased in *plexA* heterozygous flies (Supplementary Figure S3). It is therefore possible that *plexA* modification of the mutant GlyRS phenotype, and therefore the amelioration caused by Sema2A overexpression, is at least partially mediated through increased PlexB levels.

## Discussion

**Figure 5:**
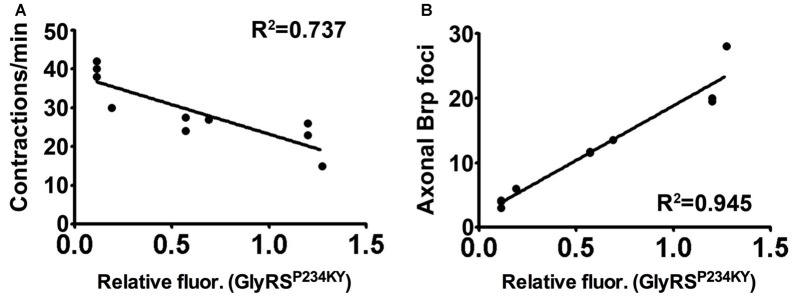
Synaptic accumulation of mutant GlyRS correlates with neuropathology. **(A,B)** Linear regression analyses identifying correlations between synaptic mutant GlyRS build-up with both muscular contractions (**A**, *r*^2^ = 0.727; *P* = 0.0015) and axonal Brp build-up (**B**, *r*^2^ = 0.945; *P* < 0.001).

We have shown that key pathologies in a *Drosophila* model of CMT2D can be modified by alterations in plexin-semaphorin signaling. *Drosophila* possess two plexins, PlexA and PlexB, which differentially bind semaphorins to provide cell-cell communication that drives axon repulsive and attractive cues. We show that mutant GlyRS expression leads to ectopic axonal branches in the peripheral nervous system, and this phenotype is modifiable by altering the levels of the *Drosophila* plexins, PlexA and PlexB. We then show that heterozygous reduction of PlexB ameliorates the lethality and neuropathology caused by *gars*^P234KY^ expression, while PlexA reduction exacerbates the same phenotypes. In addition, PlexB, but not PlexA, appears to be important for the association of mutant GlyRS with the neuromuscular synaptic membrane. Furthermore, we found that increasing expression of the secreted PlexB ligand, Sema2a, but not the PlexA ligand, Sema1a, can also supress the mutant GlyRS binding and motor defects. Finally, we show that the fluctuations in mutant GlyRS build-up at the NMJ, caused by manipulation of plexin-semaphorin components, correlates significantly with the pathological features identified in the CMT2D model (Figures 5A,B). Taken together, these data highlight the importance of plexin-semaphorin signaling in the regulation of mutant GlyRS toxicity and accumulation, and the role of the PlexB-Sema2a on mutant GlyRS synaptic build-up.

In both invertebrates and vertebrates, plexin signaling drives the attractive and repulsive signals required for high-fidelity nerve-nerve, nerve-muscle and nerve-glia interactions (Winberg et al., 1998; Hu et al., 2001; Ayoob et al., 2006; Berke and Keshishian, 2011; Roh et al., 2016; Syed et al., 2016). Although CMT2D is thought to be primarily degenerative in nature, neurodevelopment and maturation defects are observed in invertebrate (Grice et al., 2015b) and vertebrate (Sleigh et al., 2014b, 2017a) models, with motor neuron migration (He et al., 2015) and sensory neuron branching defects seen in mutant mouse embryos (Sleigh et al., 2017a). It has not been established how the neurodevelopmental defects arise and whether they are a precursor to pathology, or an epiphenomenon. Our data in *Drosophila* indicate that mutant GlyRS interferes with the fidelity of axon guidance pathways that are partly governed by both PlexA and PlexB signaling. As mutant GlyRS expression both phenocopies and modifies the *plexA* and *plexB* axonal branching defects, we suggest that the ectopic branching phenotype arises from mutant GlyRS interaction and interference with canonical plexin signaling. Importantly, however, whilst we found opposing effects of PlexA and PlexB on toxicity phenotypes and associated pathology, the same neurodevelopment defects were exacerbated with reduction of either plexin. Hence, the data suggest that axonal guidance defects are de-coupled from the neurodegenerative pathology, mirroring the developmental switching in sensory neuron identity observed in *Gars* mice that appears to be independent from the progressive peripheral nerve degeneration (Sleigh et al., 2017a).

In this study, we use the larval NMJ to examine the interaction between the plexin-semaphorin components and mutant GlyRS toxicity. However, unravelling the complete contribution of plexin-semaphorin signaling to CMT2D, in both the peripheral and central nervous systems, is made difficult by the complexity of the temporally and spatially diverse functions mediated through both plexin types (Neufeld et al., 2011; Roh et al., 2016). For example, *Drosophila* PlexA has been shown to drive repulsive axon guidance cues in the embryo, whilst PlexB can initiate either repulsive or attractive cell-cell interactions depending on the cellular environment (Winberg et al., 1998; Berke and Keshishian, 2011; Wu et al., 2011; Jeong et al., 2012). In addition, the repulsive and attractive actions are mediated though different ligands and interact with different downstream effectors (Winberg et al., 1998; Hu et al., 2001; Ayoob et al., 2006; Bates and Whitington, 2007; Berke and Keshishian, 2011; Cho et al., 2012; Roh et al., 2016). Plexin A binds strongly to the Sema1 class of semaphorins, while Plexin B binds to the Sema2 class (Winberg et al., 1998; Ayoob et al., 2006; Bates and Whitington, 2007). The plexin-semaphorin interactions occur in both competitive and cooperative processes, and can involve both forward and reverse signaling cascades. We show that overexpression of *Sema2a* in larval muscle suppresses the motor and neuronal phenotypes observed when mutant GlyRS is expressed. In contrast, there is no change in the phenotypic readouts when the class 1 semaphorin, Sema1A, is overexpressed. Accordingly, PlexB reduction, unlike PlexA, was able to partly rescue the mutant GlyRS lethality. We thus propose that PlexB is required for the build-up of mutant GlyRS at the NMJ, and ultimately the toxicity caused by expression of *gars*^P234KY^. Mutant GlyRS could be competing with Sema2A binding at the larval NMJ, and interfere with other membrane-bound or secreted factors associated with different neuronal cell types, or at different developmental time points. It is likely that the *plexB* heterozygote fly did not produce a complete rescue of mutant GlyRS because *plexB* is still expressed, albeit at a reduced level. Consistent with a role for PlexB-mutant GlyRS binding in synaptic accumulation, the overexpression of Sema2A, particularly in the reduced *plexB* fly, led to further reduction in GlyRS build-up and an additive improvement in disease phenotypes, presumably by out-competing mutant GlyRS.

Within the cell, GlyRS charges glycine to its cognate tRNA; however, in a non-canonical tumur-defense capacity, GlyRS also binds to the extracellular receptor protein K-cadherin (Park et al., 2012). Moreover, GlyRS functions in neddylation by interacting with NEDD8 (Mo et al., 2016). A non-canonical neuronal receptor protein has not yet been reported for wild-type GlyRS, but mutant GlyRS has been shown to interact with multiple anomalous targets (He et al., 2015; Sleigh et al., 2017a). Our data suggest that Plexin B may either be an additional, or fly-specific, aberrant target, or an indirect modifier of the interactions that drive the association of mutant GlyRS with the presynaptic membrane. In mice, mutated GlyRS erroneously binds to Nrp1 and this interaction competes with the binding of the endogenous ligand VEGF (He et al., 2015). The data we present here are similar in that an anomalous interaction between mutated GlyRS and PlexB competes with the endogenous ligand, Sema2A. Indeed, VEGF treatment partially rescues motor function of the CMT2D mice and Nrp1 heterozygosity exacerbates it (He et al., 2015), while reduced expression of VEGF in wild-type mice has been shown to contribute to motor neuron degeneration (Oosthuyse et al., 2001). These findings argue that in both fly and mouse, secreted mutant GlyRS interacts with neuronally-expressed transmembrane proteins involved in signaling. The downstream consequences of aberrant binding remain to be elucidated; however, the amelioration of the phenotype by reducing plexin B indicates that loss of Sema2A-PlexB signaling is not the mechanism. Instead, the strong correlation we observed between presynaptic mutant GlyRS accumulation and neuromuscular dysfunction suggests that the former is an important part of the pathogenic process (Figure 5).

We have suggested that Plexin B may either be an anomalous target for mutant GlyRS, or an indirect modifier of the interactions that drive the association of mutant GlyRS with the presynaptic membrane. Our data also suggest that PlexA may further modify the phenotypes indirectly through its physiological antagonism of PlexB-driven processes. Interactions of this nature have been noted previously; for example, PlexA has been shown to partially rescue the PlexB mutant, but PlexB did not reciprocally rescue PlexA mutants (Ayoob et al., 2006). We have shown that the presence of the plexA heterozygous mutation eliminates the mutant *gars* phenotype amelioration conferred by Sema2A overexpression, suggesting that PlexA may act independently on mutant GlyRS toxicity at the NMJ, and downstream of PlexB. Beyond this, as multiple semophorin-plexin interactions control nerve-nerve, nerve-muscle, and nerve-glia communication (Ayoob et al., 2006; Bates and Whitington, 2007; Yu et al., 2010; Syed et al., 2016), a range of parallel mechanisms may cause defects that ultimately lead to broader changes in the set-up and maintenance of neuronal circuits and synapses of the animal. Moreover, additional signaling pathways may drive parallel neuroprotective actions within the nervous system, and therefore titre mutant GlyRS binding and toxicity further.

In summary, we have shown that mutants GlyRS expression interferes with plexin-mediated axonal branching, and that the alteration of plexin-semaphorin signaling can modify mutant GlyRS lethality, neuromuscular dysfunction, and neuropathology. This toxic gain-of-function, and alteration of neurodevelopmental pathways, highlights mechanisms analogous to a mammalian model of the disease. Our findings provide additional evidence of a new disease paradigm in CMT2D, which provides further explanation for the selective effect on the nervous system of mutations in a widely expressed protein.

## Author Contributions

SJG, JNS and MZC designed the experiments and wrote the article. SJG and JNS performed the experiments and analyzed the data. All authors have approved submission of this work.

## Conflict of Interest Statement

The authors declare that the research was conducted in the absence of any commercial or financial relationships that could be construed as a potential conflict of interest.
