# Supplementary material for: Plexin-Semaphorin Signaling Modifies Neuromuscular Defects in a Drosophila Model of Peripheral Neuropathy
Source: Front Mol Neurosci. 2018 Feb 22;11:55. doi: 10.3389/fnmol.2018.00055 (PMC5827687; doi:10.3389/fnmol.2018.00055)
Supplement: Supplementary file 1 [file Image_1.pdf]

Plexin-semaphorin signalling modifies neuromuscular defects in a Drosophila model of peripheral neuropathy

Stuart J. Grice[1], James N. Sleigh[2], M. Zameel Cader[1,3*]

Affiliations:

[1] MRC Functional Genomics Unit, Department of Physiology, Anatomy and Genetics, University of Oxford, South Parks Road, Oxford OX1 3PT, UK.

[2] Sobell Department of Motor Neuroscience and Movement Disorders, Institute of Neurology, University College London, London WC1N 3BG, UK.

[3] University of Oxford, Nuffield Department of Clinical Neurosciences, John Radcliffe Hospital, Oxford OX3 9DU, UK.

* Correspondence to: M. Zameel Cader
Email: zameel.cader@ndcn.ox.ac.uk
Tel: +44 (0) 1865 231858
Fax: +44 (0) 1865 272420

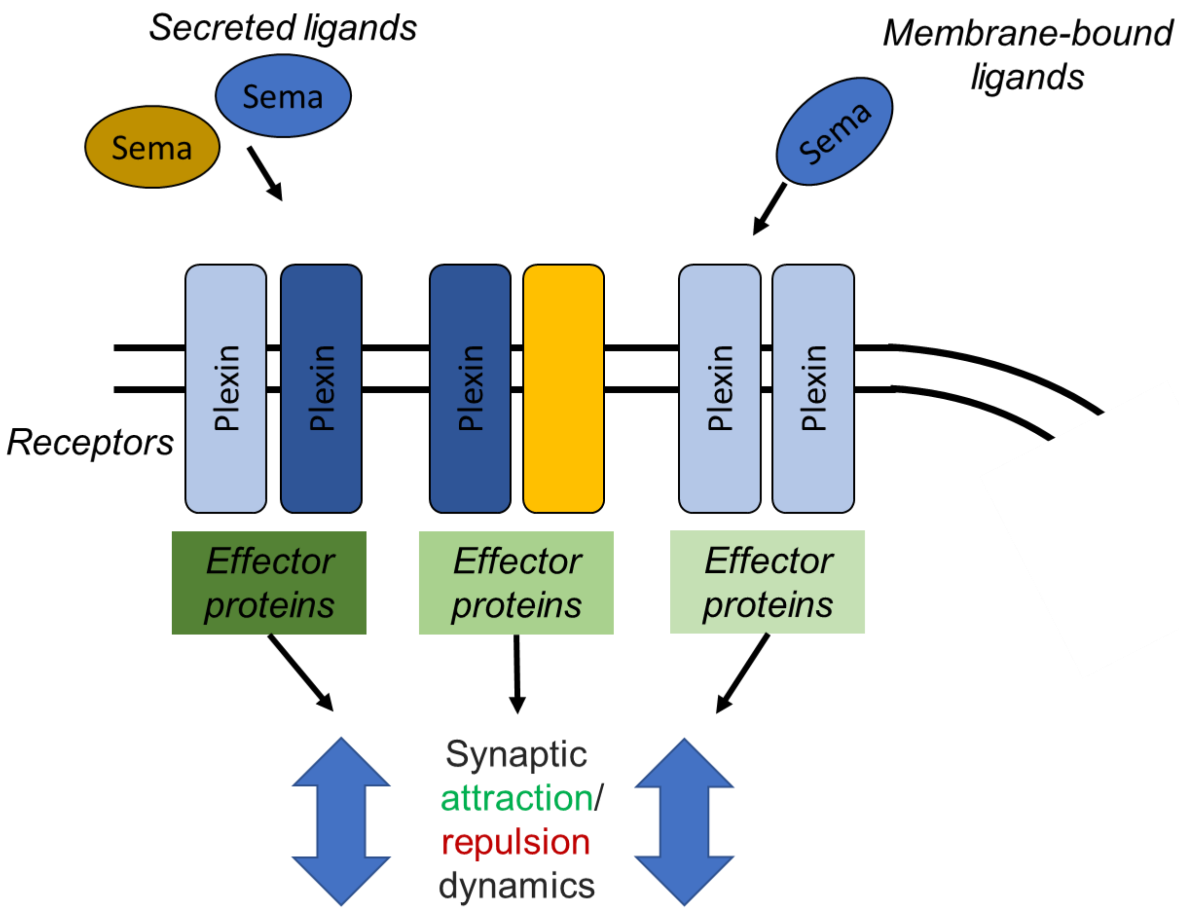

Figure S1. Plexin-semaphorin signalling. In vertebrates and invertebrates, plexins are transmembrane receptors that bind to secreted and membrane-bound semaphorins. Plexins act in conjunction with co-receptors, such as neuropilins (yellow protein), to drive signals that mediate repulsive and attractive cues at the synapse. The collaboration of different combinations of ligands, plexins, co-receptors, and downstream effector proteins impact the dynamics of axonal attraction and repulsion.

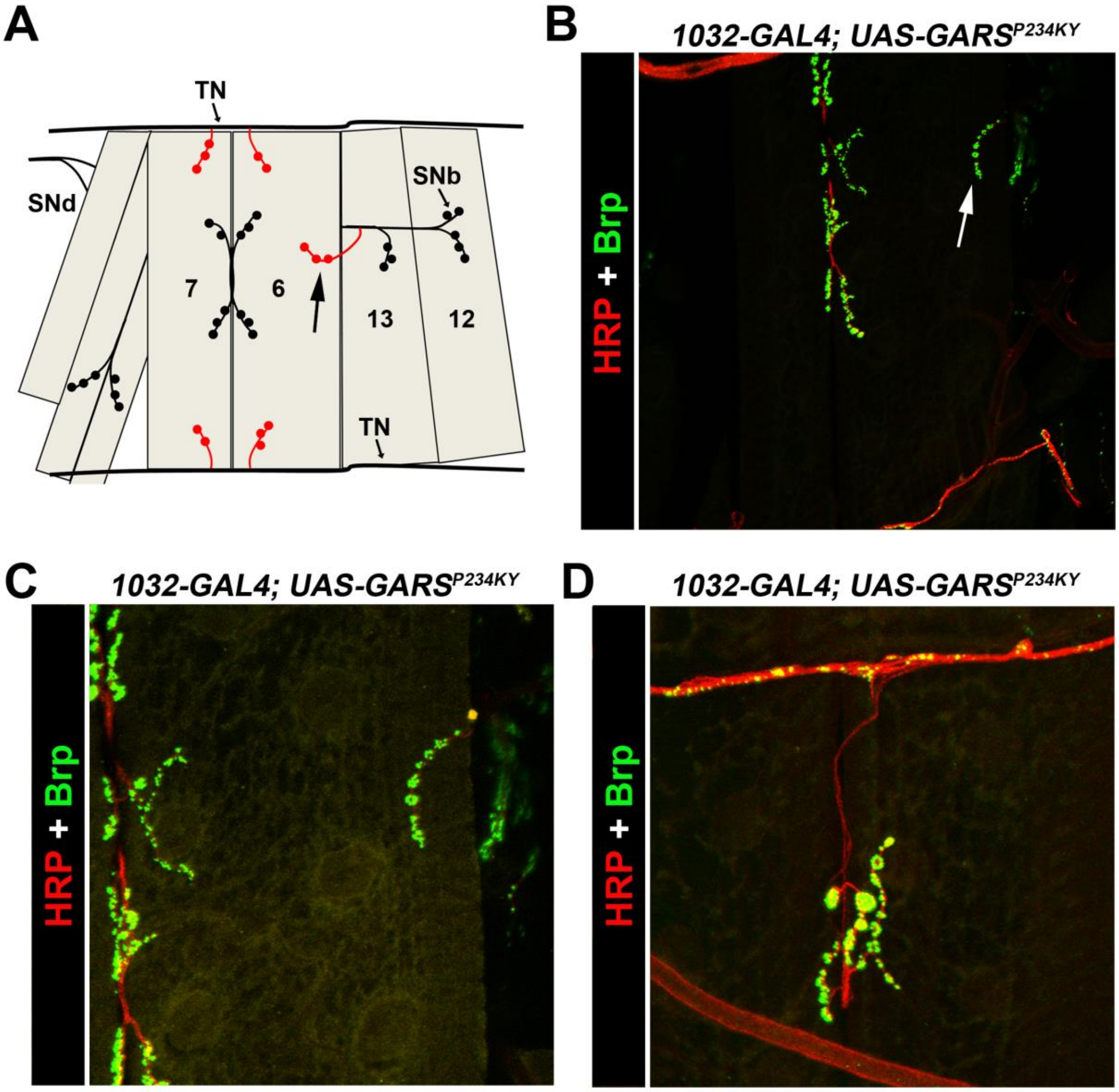

Figure S2. Ectopic synaptic contacts from segmental nerve b in mutant *gars* flies. (A) Schematic of the larval ventral body wall muscles in one hemisegment, showing the transverse nerve (TN), and branches of segmental nerves b (SNb) and d (SNd). The ectopic synaptic contacts often observed on muscles 6 and 7 in *gars*^{P234KY} flies are shown in red. This schematic is the same as in Fig. 1A. **(B)** Ubiquitous expression (*1032-GAL4* driver) of mutant *gars* leads to ectopic synaptic contacts from the SNb onto muscle 6. Large arrows in both panels highlight ectopic branches of the SNb. **(C-D)** Zoomed images of the ectopic axons and synaptic contacts observed from the SNb (**C**; zoom from **B**) and the TN (**D**; zoom from Figure 1C) when *gars*^{P234KY} is expressed ubiquitously, Scale bars = 10 µm

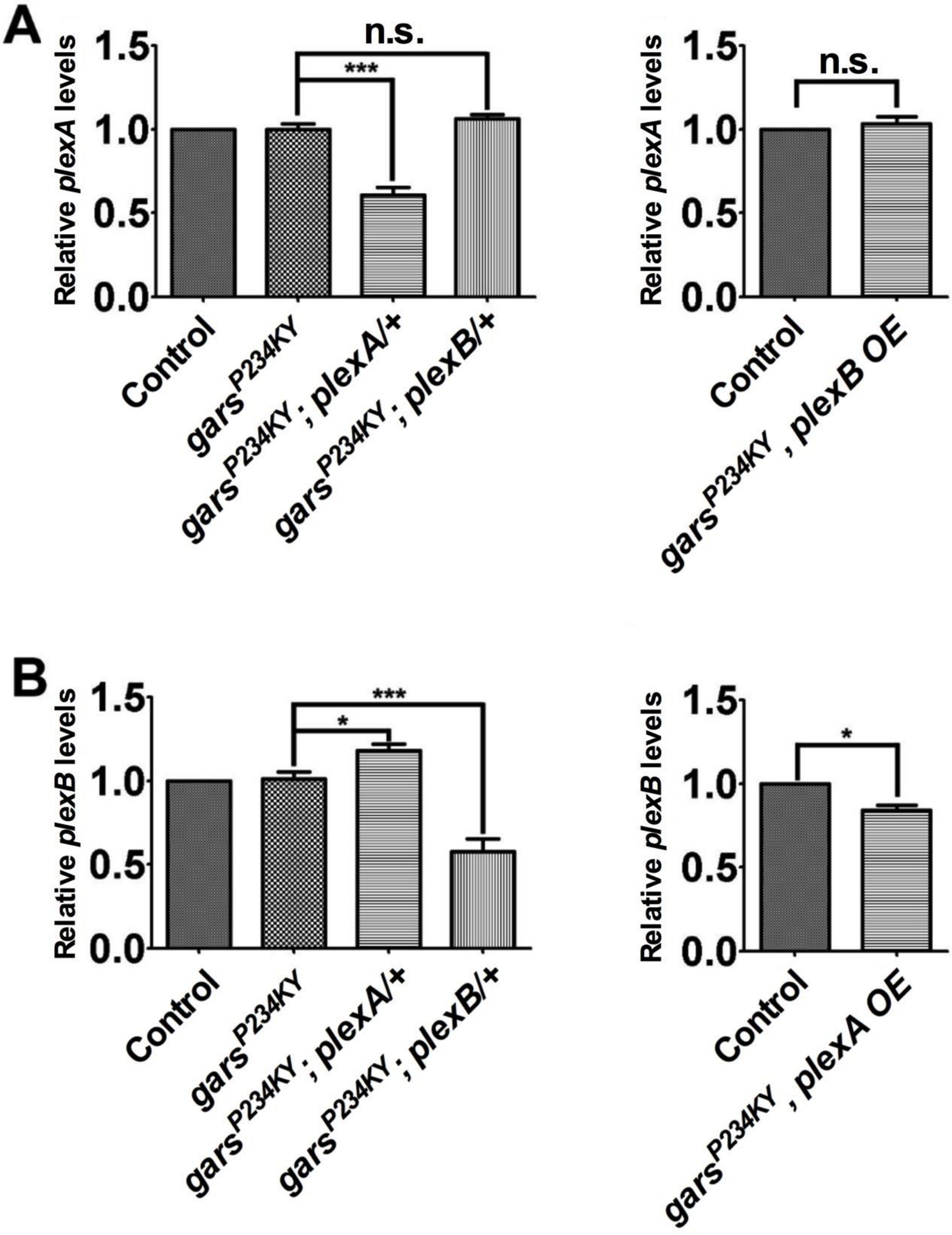

Figure S3. ***plexA*** **and** ***plexB*** **expression in GlyRS-plexin interaction mutant** ***Drosophila*** **backgrounds.** (**A, B**) qPCR analyses of *plexA* (A) and *plexB* (B) expression in larval ventral nerve cords. Ubiquitous mutant *gars* expression has no impact on *plexA* or *plexB* RNA levels (A, B, left). *gars*^{P234KY} flies on a heterozygous *plexA* background have the expected reduction in *plexA* expression, while *plexA* levels remain constant in *gars*^{P234KY}*; plexB/+* flies (A, left). In contrast, *plexB* levels are significantly increased in the *gars*^{P234KY}*; plexA/+* flies (B, left). Accordingly, overexpression of *plexB* has no effect on *plexA* levels (A, right), whereas *plexA* overexpression leads to a reduction in *plexB* (B, right). *rp42* was used as the reference gene. * $P < 0.05$, *** $P < 0.001$ Dunn's/Bonferroni's multiple comparison test (left panels) or *t*-test (right panels). n.s., not significant.

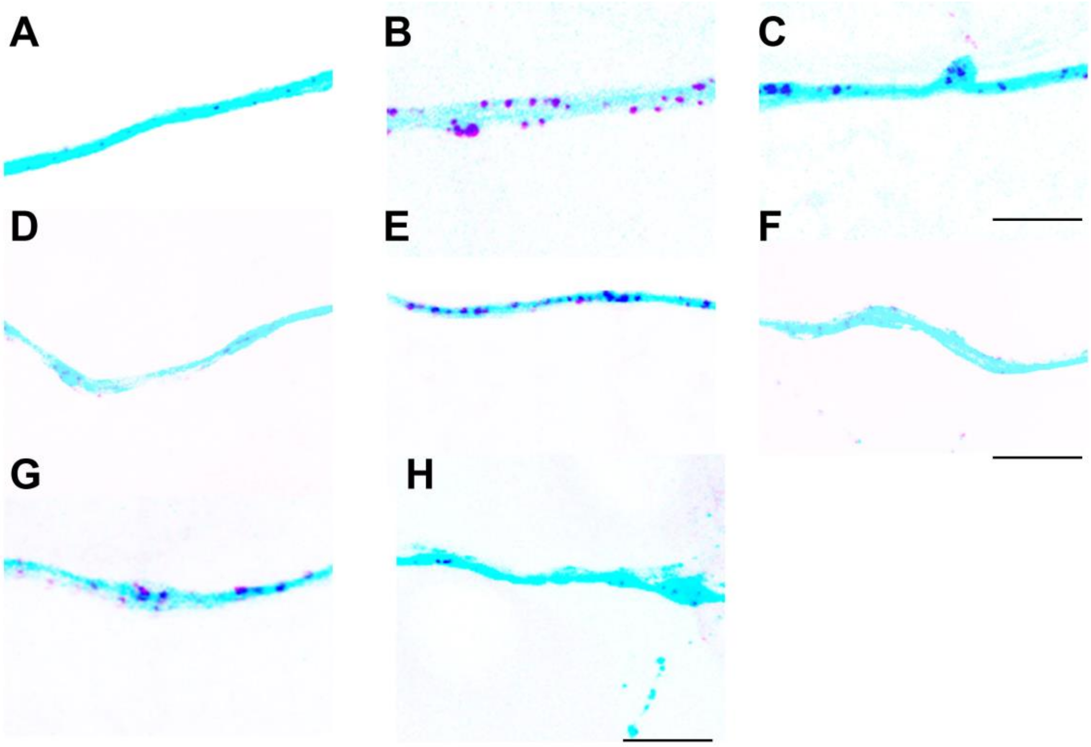

Figure S4 *plexA* and *plexB* dosage changes modify GlyRS^{P234KY}-associated Brp axonal localisation. (A-C) Zoomed images of the TN showing increased axonal Brp compared to control **(A**; zoom from Figure 3A**)** upon ubiquitous **(B**; zoom from Figure 3B**)** and muscle-specific (**C**; zoom from Figure 3C) *gars*^{P234KY} expression. (**D-G**) Representative images showing axonal Brp in mutant gars expressing flies with *PlexB* reduction (**D**), *PlexB* overexpression (**E**), *PlexA* over expression (**F**), *PlexA* reduction (**G**), and Sema2a and *gars*^{P234KY} expression (H). Scale bars = 10 µm.
